# Auditory Attention Detection via Cross-Modal Attention

**DOI:** 10.3389/fnins.2021.652058

**Published:** 2021-07-21

**Authors:** Siqi Cai, Peiwen Li, Enze Su, Longhan Xie

**Affiliations:** Shien-Ming Wu School of Intelligent Engineering, South China University of Technology, Guangzhou, China

**Keywords:** auditory attention, attention mechanism, cocktail party, cross-modal, EEG

## Abstract

Humans show a remarkable perceptual ability to select the speech stream of interest among multiple competing speakers. Previous studies demonstrated that auditory attention detection (AAD) can infer which speaker is attended by analyzing a listener's electroencephalography (EEG) activities. However, previous AAD approaches perform poorly on short signal segments, more advanced decoding strategies are needed to realize robust real-time AAD. In this study, we propose a novel approach, i.e., cross-modal attention-based AAD (CMAA), to exploit the discriminative features and the correlation between audio and EEG signals. With this mechanism, we hope to dynamically adapt the interactions and fuse cross-modal information by directly attending to audio and EEG features, thereby detecting the auditory attention activities manifested in brain signals. We also validate the CMAA model through data visualization and comprehensive experiments on a publicly available database. Experiments show that the CMAA achieves accuracy values of 82.8, 86.4, and 87.6% for 1-, 2-, and 5-s decision windows under anechoic conditions, respectively; for a 2-s decision window, it achieves an average of 84.1% under real-world reverberant conditions. The proposed CMAA network not only achieves better performance than the conventional linear model, but also outperforms the state-of-the-art non-linear approaches. These results and data visualization suggest that the CMAA model can dynamically adapt the interactions and fuse cross-modal information by directly attending to audio and EEG features in order to improve the AAD performance.

## 1. Introduction

Humans have the ability to pay selective attention to one speaker in a multispeaker environment, also called the “cocktail party scenario” (Cherry, 1953; Haykin and Chen, 2005). However, people with hearing loss find that such situations are particularly difficult. Modern hearing aids have been developed to produce a better experience by reducing background noise and increasing speech intelligibility, such as noise reduction system and directional microphone (Wu et al., 2019). However, existing approaches usually fail in the cocktail-party situation and many hearing aid users complain about the difficulty of following a target speaker in the presence of noisy and other competing speech streams (Chung, 2004). Recent developments in the field of neuroscience have shown that it is possible to decode the auditory attention in a multi-talker environment from brain signals (Ding and Simon, 2012; Mesgarani and Chang, 2012). This is known as auditory attention detection (AAD). The development of AAD opens up new opportunities to the cognitive control of auditory prostheses, such as hearing aids and cochlear implants.

EEG provides a non-invasive means of investigating cortical activity with high temporal resolution and is a realistic option for BCI applications. Various experiments have verified the feasibility of decoding the selective attention in a multispeaker environment using EEG (Choi et al., 2013; Mirkovic et al., 2015; O'Sullivan et al., 2015; Van Eyndhoven et al., 2016; Deckers et al., 2018; Bednar and Lalor, 2020; Cai et al., 2020, 2021; Wang et al., 2020). The decoding of selective auditory attention from non-invasive EEG signals is of interest in BCI and auditory perception research and can mainly be divided into linear and non-linear approaches. Previous approaches for decoding the attentional selection of listeners have mainly focused on linear mappings between the features of sound streams and EEG responses. More specifically, the mapping from auditory stimuli to cortical responses is typically referred to as the forward model or temporal response function (TRF) (Crosse et al., 2016; Wong et al., 2018), whereas the mapping from cortical responses to acoustic features is referred to as the backward model or stimulus-reconstruction (Fuglsang et al., 2017). Moreover, de Cheveigné et al. (2018, 2019) have proposed an alternative to both forward and backward mapping, i.e., canonical correlation analysis (CCA). However, the performance of these linear decoding approaches decreases significantly when operated at low latency settings. For instance, the accuracy of linear AAD models is fairly low (approximately 60%) over a data window with a length of 1 s, the time scale at which humans are able to switch attention from one speaker to another (Zink et al., 2017). We argue that the linear mappings approach has two deficiencies. First, its mapping and correlation evaluation process are not jointly optimized for attention detection; second, both forward and backward mapping leads to fairly low correlation values, e.g., *r* = 0.054 (O'Sullivan et al., 2015). Such low correlation scores support that linear mapping may not necessarily represent the best approach for AAD. Recently, non-linear models have been proposed to detect the attended speakers based on EEG signals to realize low-latency AAD. de Taillez et al. (2017) studied a non-linear neural network for mapping EEG signals to speech envelopes in a cocktail party scenario and showed that it outperforms the linear model baseline. Following a similar approach, convolutional neural network (CNN) models (Deckers et al., 2018; Ciccarelli et al., 2019; Cai et al., 2020; Vandecappelle et al., 2021) were studied to detect the attended speakers. However, these non-linear AAD approaches neglect valuable temporal information of EEG signals and more advanced decoding strategies are needed to realize robust real-time AAD.

In this paper, we further study a non-linear decoder for real-time AAD and develop a cross-modal attention mechanism, which is referred to as cross-modal attention-based auditory attention detection (CMAA). The CMAA model can detect auditory attention directly from enhanced audio and EEG features without the reconstruction process (e.g., without reconstructing auditory stimulus from EEG signals). The core of our proposed CMAA model is the cross-modal attention module, which can model the top-down and bottom-up modulation by dynamically assigning weights at run-time according to the input stimulus. The attention mechanism has attracted great interest and shown promising capability in a variety of related applications such as machine translation (Luong et al., 2015), image caption generation (Xu et al., 2015) and object classification (Wang et al., 2016; Guo et al., 2019). Give that the fundamental theory of the AAD model is based on the relationship between the auditory stimuli and the EEG responses elicited by these stimuli, we employ the CMAA model to dynamically modulate the interactions of EEG and audio streams in the temporal domain, analogous to how human brains selectively attend to input stimuli. Considering that brain activity is a temporally dynamic process and EEG signals are essentially non-linear time series data (Bassett and Sporns, 2017), the proposed CMAA has an advantage over CNN in capturing temporal characteristics of EEG. With the cross-modal attention mechanism, we hope to adapt EEG to audio streams by repeated reinforcement of the EEG features with those from audio, or vice versa, thus improving low-latency AAD performance.

Overall, we explored a novel CMAA approach which allows dynamic interaction between the audio and EEG features to improve the observations of the relation between auditory stimulus and EEG response. The proposed CMAA model was evaluated on a publicly available database, i.e., DTU (Fuglsang et al., 2017, 2018), which is described in detail in section 2.5. The main contributions of this study can be summarized as follows:

1) We have developed a novel framework for EEG-based AAD. The proposed CMAA framework integrates the cross-modal attention mechanism into an AAD decoder to capture the correlation between auditory stimuli and EEG responses in order to improve the AAD performance.
2) CMAA framework consists of bi-directional cross-modal attention, which transforms both the auditory stimulus and the EEG response. Compared to previous methods based on the forward or backward models for linear mapping, CMAA maximizes the mutual information and supports AAD decoder that yields higher classification accuracy.
3) We conducted experiments on the DTU database, and the experimental results indicated the proposed CMAA method can realize reliable detection of auditory attention in low latency settings under real-world reverberant conditions.

## 2. Materials and Methods

**Figure 1:**
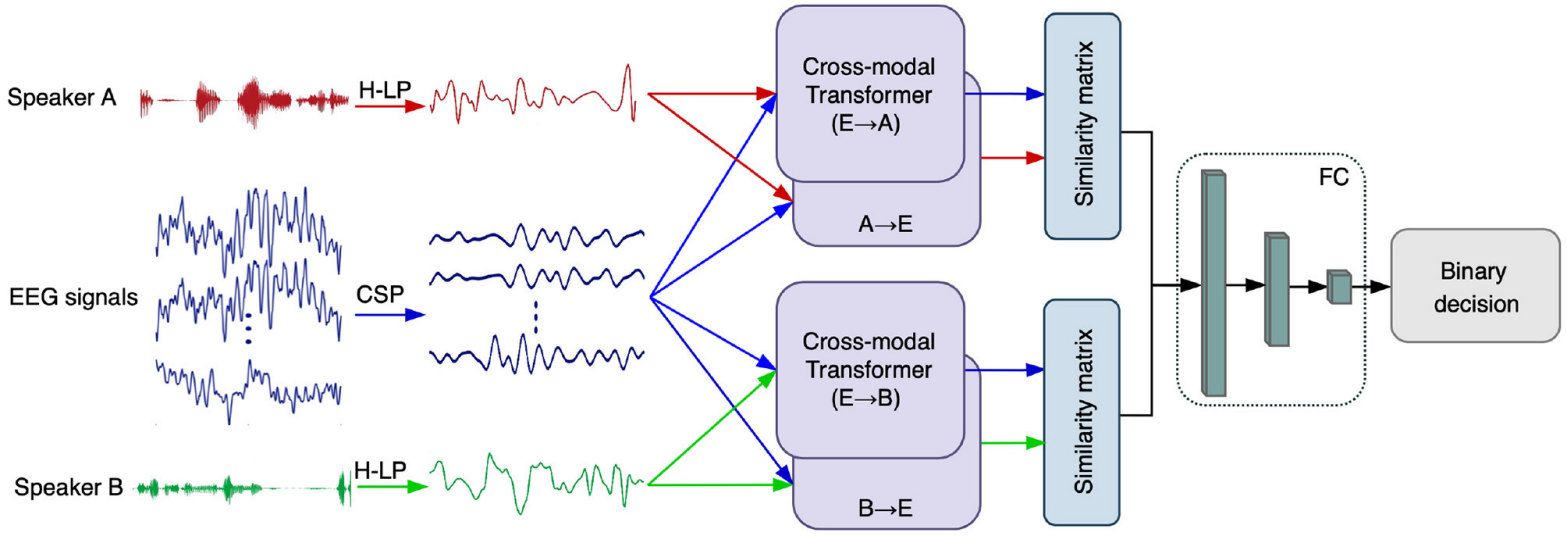
A schematic diagram of our proposed cross-modal attention-based AAD network (CMAA). First, we employ CSP algorithm for EEG enhancement and H-LP to improve the speech envelope extraction process. Then, the cross-modal attention, which takes EEG and audio features as input, is the core component of the CMAA for dynamic interaction. The proposed CMAA architecture tackles all pairs of modalities with the cross-modal attention module, including EEG signals → audio of speaker A (E → A), EEG signals → audio of speaker B (E → B), audio of speaker A → EEG signals (A → E) and audio of speaker B → EEG signals (B→ E). Finally, the cross-modal similarity are computed and compared to determine the attended speaker. Here, the audio streams of speaker A and speaker B are denoted in red and green, while the EEG signals are denoted in blue.

AAD is usually formulated as a binary classification problem in a two-speaker scenario (de Taillez et al., 2017; Deckers et al., 2018; Vandecappelle et al., 2021). First, the CSP method was used for discriminative feature extraction of the original EEG signals. Meanwhile, we apply an auditory-inspired linear filter bank and power-law compression to improve the speech envelope extraction process (Biesmans et al., 2016), which is denoted as H-LP and is described in detail in section 2.6. Then, we employ the cross-modal attention module to adjust the interactions of EEG and auditory stimuli. The advanced feature extraction and attention mechanisms are expected to improve the separation between the EEG signals of opposite attention, and obtain optimal interactions between the EEG signals and auditory stimuli. Finally, a similarity matrix is used to evaluate the correlation coefficient (cosine similarity) between the EEG responses and the attended and unattended auditory stimulus, respectively. The speaker with greater correlation is chosen as the attended speaker, while the unattended speaker is identified as the speaker with the weaker correlation. The overall CMAA architecture is illustrated in Figure 1, and is explained in detail below.

### 2.1. Common Spatial Pattern

Considering the low signal-to-noise ratio of raw EEG data, we applied the common spatial pattern (CSP) algorithm for EEG signal enhancement (Ramoser et al., 2000; Pfurtscheller and Neuper, 2001). Previous studies have demonstrated that classification performed on the CSP features generally yields better accuracy in motor imagery BCI systems (Blankertz et al., 2007a; Zhang et al., 2017). Moreover, CSP method shows potential for the improvement of EEG-based AAD performance, as has been demonstrated in our pilot study (Cai et al., 2020). CSP could find a projection matrix composing of several pairs of space filtering vector. And the multi-channel EEG signals are projected into a new space through the projection matrix so that the variance of one class is maximized and the other is minimized (Pfurtscheller and Neuper, 2001; Blankertz et al., 2007b). Considering that we formulate a two-speaker AAD problem as a binary classification task, we expect the CSP algorithm to be effective in discriminating the EEG signals corresponding to two opposite speakers.

The principle of CSP is find an optimal spatial filter with diagonalization calculation to project the EEG signals into a new feature space and maximize the variance between the classes. Assume that we have two sets of EEG data, *G*_*A*_ and *G*_*B*_, recorded for two attended speakers A and B, respectively. Each set of EEG data can be represented as a multichannel evoked response matrix with *M* × *S* dimensions, where *M* is the number of the channels and *S* is the number of the samples from each channel. The composite covariance matrix and its eigenvalue decomposition are given by

(1) $$\begin{array}{l} C=C_{A}+C_{B}\\ \;=\frac{G_{A}G_{A}^{T}}{tr\left(G_{A}G_{A}^{T}\right)}+\frac{G_{B}G_{B}^{T}}{tr\left(G_{B}G_{B}^{T}\right)}\\ \;=Z\psi Z^{T} \end{array}$$

where *C*_*A*_ and *C*_*B*_ are the covariance matrices of *G*_*A*_ and *G*_*B*_. *tr*(·) is sum of elements on the main diagonal of a matrix as the trace of the matrix. *T* denotes the transpose operator. *Z* is a matrix of normalized eigenvectors with corresponding matrix of eigenvalues, ψ.

The whitening transformation matrix

(2) $$\begin{array}{l} P=\sqrt{\psi^{-1}}Z^{T} \end{array}$$

transforms the covariance matrices as

(3) $$\begin{array}{l} C_{A}\prime =PC_{A}P^{T},C_{B}\prime =PC_{B}P^{T} \end{array}$$

where *C*_*A*_′ and *C*_*B*_′ share common eigenvectors, and the sum of corresponding eigenvalues for the two matrices are always one, such that

(4) $$\begin{array}{l} C_{A}\prime =U\lambda_{A}U^{T},C_{B}\prime =U\lambda_{B}U^{T},\lambda_{A}+\lambda_{B}=I \end{array}$$

where *I* is the identity matrix. *U* and λ respectively denote the matrix of eigenvectors and the diagonal matrix of eigenvalues.

Thus, we can obtain the CSP projection matrix *W* = *U*^*T*^*P* with the eigenvectors from the decomposition. And the EEG features after spatial filtering can be expressed as:

(5) $$\begin{array}{l} F_{i}=WG_{i} \end{array}$$

where {*F*_*i*_ : *i* ∈ {*A, B*}} denotes the resulting CSP-enhanced EEG features.

### 2.2. Cross-Modal Attention

The fundamental theory of the AAD model is the relationship between the auditory stimuli and the cortical responses elicited by these stimuli. From our perspective, the interaction between auditory stimulation and EEG responses can be formulated as a cross-modal problem. Specifically, cross-modal attention can dynamically adapt the streams from one modality to another and correlate meaningful elements across these two modalities (Peng et al., 2017; Ji et al., 2020). In addition, previous studies (Anderson et al., 2018; Yuan and Peng, 2019; Paraskevopoulos et al., 2020; Xu et al., 2020) have shown that the cross-modal attention mechanism can achieve better performance than the state-of-the-art methods in the multimedia field. Therefore, we develop a model with cross-modal attention to fully explore the correlations between audio and EEG signals, so as to solve the AAD problem in this study.

As stated in previous studies (Vaswani et al., 2017; Paraskevopoulos et al., 2020), the attention function can be described as mapping a query and a set of key/value pairs to an output, where the query, keys, values, and output are vectors. The output is computed as a weighted sum of the values, where the weight assigned to each value is computed by a compatibility function of the query with the corresponding key. For cross-modal attention, assume two modalities α and β, with two streams from each of the modalities denoted by $X_{\alpha }\in {\mathbb{R}}^{T_{\alpha }\times d_{\alpha }}$ and $X_{\beta }\in {\mathbb{R}}^{T_{\beta }\times d_{\beta }}$, respectively. Here, *T* denotes the duration of each stream and *d* denotes the feature dimension. We define the query as *Q*_α_ = *X*_α_*W*_*Q*_α__, and the key and the value as *K*_β_ = *X*_β_*W*_*K*_β__ and *V*_β_ = *X*_β_*W*_*V*_β__, respectively. Here, the projections are the weight matrices $W_{Q_{\alpha }}\in {\mathbb{R}}^{d_{\alpha }\times d_{k}}$, $W_{K_{\beta }}\in {\mathbb{R}}^{d_{\beta }\times d_{k}}$, and $W_{V_{\beta }}\in {\mathbb{R}}^{d_{\beta }\times d_{v}}$. The output from the cross-modal attention layer is represented by **O**_β→α_ and is computed as:

(6) $$\begin{array}{l} {\text{O}}_{\beta \to \alpha }=CM\left(Q_{\alpha },K_{\beta },V_{\beta }\right)\\ \;=softmax\left(\frac{Q_{\alpha }K_{\beta }^{T}}{\sqrt{d_{k}}}\right)V_{\beta }\\ \;=softmax\left(\frac{X_{\alpha }W_{Q_{\alpha }}W_{K_{\beta }}^{T}X_{\beta }^{T}}{\sqrt{d_{k}}}\right)X_{\beta }W_{V_{\beta }} \end{array}$$

where the ${\text{O}}_{\beta \to \alpha }\in {\mathbb{R}}^{d_{\alpha }\times d_{k}}$ has the same length as *Q*_α_, and $softmax\left(\cdot \right)\in {\mathbb{R}}^{T_{\alpha }\times T_{\beta }}$. $\sqrt{d_{k}}$ is the scaling factor. Specifically, the scaled softmax is the score matrix on the values, i.e., the attention map, which reflects the relationship between the two modalities. **O**_β→α_ is the weighted representation of *V*_β_.

Considering that the EEG data were collected from the subject while he/she listened to two competing speakers and was instructed to attend to one particular speaker in the AAD tasks, the proposed CMAA architecture must handle all pairs of modalities with the cross-modal attention module. As shown in Figure 1, the CMAA model consists of two directions: EEG → audio (backward direction) and audio → EEG (forward direction). Specifically, for EEG → audio attention, which is referred to as E2A attention, the model attends to EEG signals according to each audio component and then determines the importance of the audio components to the EEG by comparing each audio component to the corresponding attended EEG vector. E2A attention consists of two pairs of modalities: EEG signals → audio of speaker A and EEG signals → audio of speaker B. For audio → EEG attention direction, which is referred to as A2E attention, the model attends to the audio components for the EEG signals and determines the importance of the EEG components for the audio attention vector. A2E attention also treats two pairs of modalities: the audio of speaker A → EEG signals and audio of speaker B → EEG signals.

**Figure 2:**
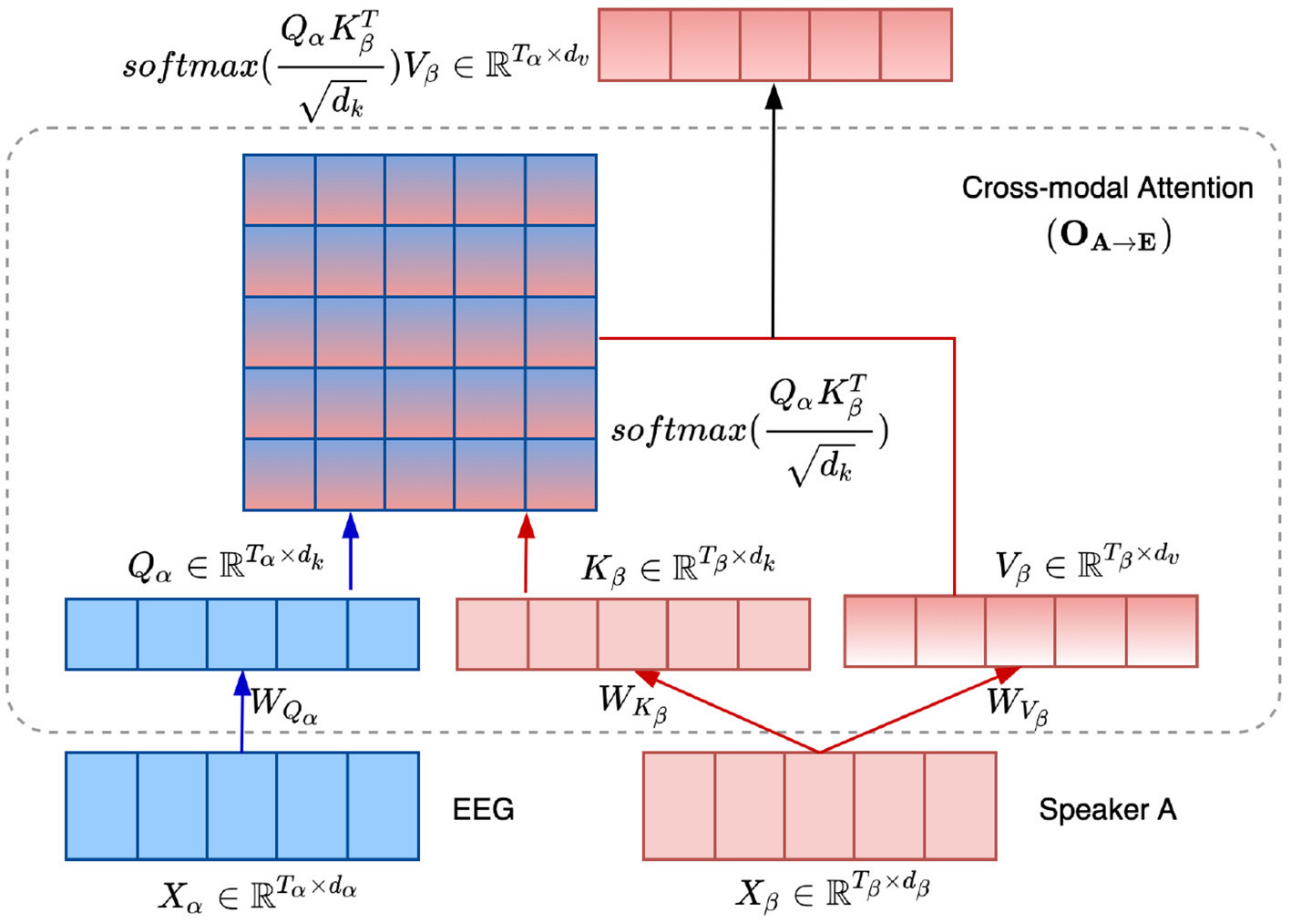
Illustration of the cross-modal attention mechanism between the audio of speaker A and EEG signals, i.e., **O**_*A*→*E*_.

Taking the audio of speaker A → EEG signals as an example, the detailed architecture of the cross-modal attention (**O**_*A*→*E*_) is depicted in Figure 2. Specifically, we employ the audio of speaker A as the β modality, while CSP-enhanced EEG features as the α modality in equation 6. Thus, the cross-modal attention mechanism adaptively adjusts the weights of the audio components and emphasizes the most informative components of the audio signal based on the EEG attention vector, realizing the forward direction AAD. Moreover, the backward direction AAD is realized with the E2A attention, where EEG is the β modality and audio is the α modality.

### 2.3. Overall Architecture for Cross-Modal Transformer

**Figure 3:**
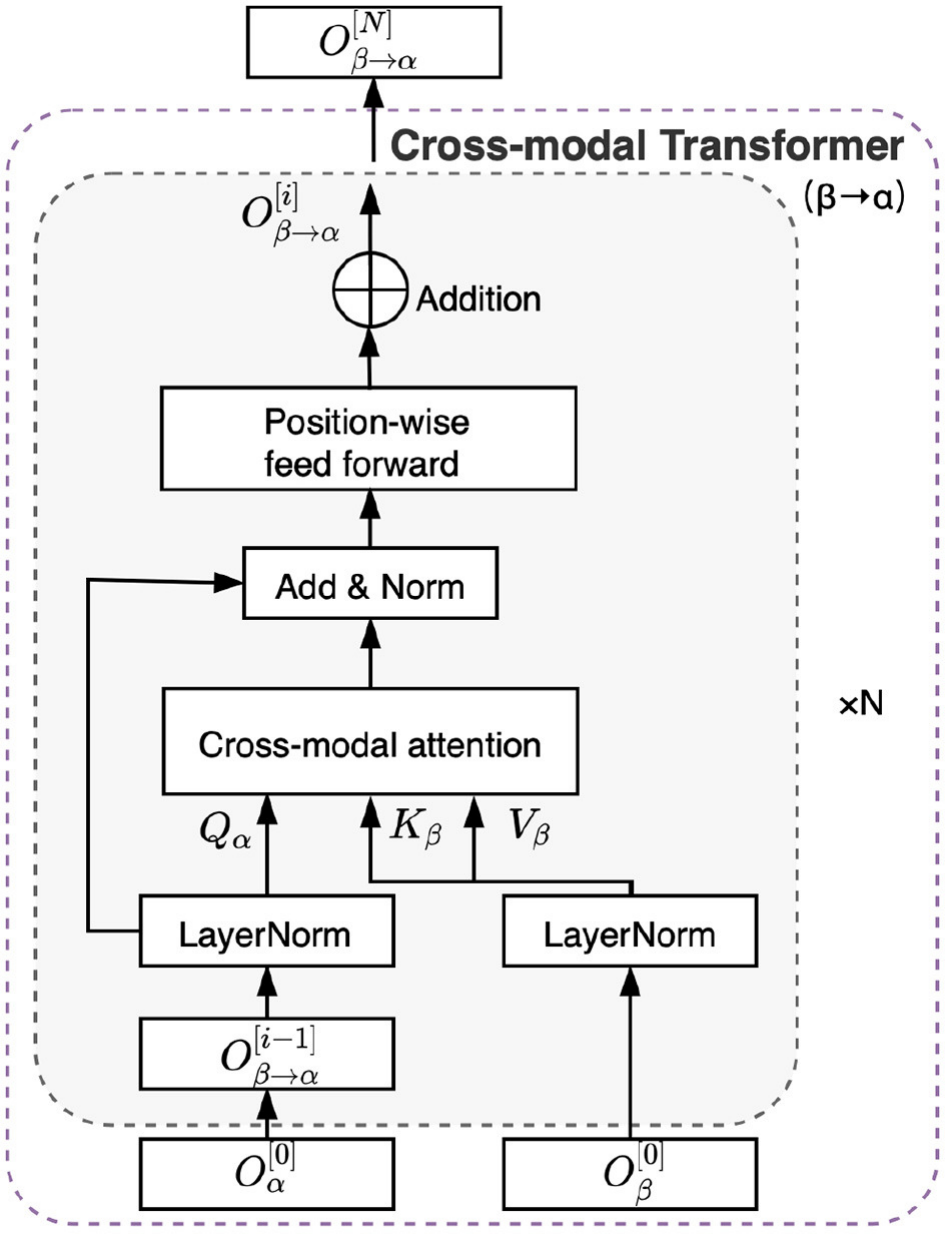
Illustration of the cross-modal transformer architecture between two time-series from modality α and β.

Based on the cross-modal attention mechanism, we developed the cross-modal transformer, which is based on the transformer architecture (Vaswani et al., 2017), as shown in Figure 3. Briefly, the cross-modal transformer consists of N layers. The first operation in each layer is a cross-modal attention block, as illustrated in detail in section 2.2. The second operation is a positionwise feed-forward layer block that consists of two linear transformations with a rectifying linear unit (ReLU) activation in between (Vaswani et al., 2017). Therefore, the cross-modal transformer computes feed-forwardly for i = 1,..., N layers, as follows:

(7) $$\begin{array}{l} {\text{O}}_{\beta \to \alpha }^{\left[0\right]}={\text{O}}_{\alpha }^{\left[0\right]}\\ {\bar{\text{O}}}_{\beta \to \alpha }^{\left[i\right]}={\text{C}\text{M}}_{\beta \to \alpha }^{\left[i\right]}\left(LayerNorm\left({\text{O}}_{\beta \to \alpha }^{\left[i-1\right]}\right),LayerNorm\left({\text{O}}_{\beta }^{\left[0\right]}\right)\right)\\ \;+LayerNorm\left({\text{O}}_{\beta \to \alpha }^{\left[i-1\right]}\right)\\ {\text{O}}_{\beta \to \alpha }^{\left[i\right]}={\left(LayerNorm\left({\bar{\text{O}}}_{\beta \to \alpha }^{\left[i\right]}\right)\right)}^{FF}+LayerNorm\left({\bar{\text{O}}}_{\beta \to \alpha }^{\left[i\right]}\right) \end{array}$$

where *LayerNorm* denotes layer normalization (Ba et al., 2016). It is a recently introduced method for normalizing the activities of neurons in deep neural networks to help stabilize training and boost model convergence. ${\left(LayerNorm\left({\bar{\text{O}}}_{\beta \to \alpha }^{\left[i\right]}\right)\right)}^{FF}$ is transformed by the positionwise feed-forward block and can be computed as follows:

(8) $$\begin{array}{r} {\left(LayerNorm\left({\bar{\text{O}}}_{\beta \to \alpha }^{\left[i\right]}\right)\right)}^{FF}=max(0,(LayerNorm({\bar{\text{O}}}_{\beta \to \alpha }^{\left[i\right]})){\text{W}}_{1}\\ +{\text{b}}_{1}){\text{W}}_{2}+{\text{b}}_{2} \end{array}$$

where **W**_1_ and **W**_2_ denote the first and second linear projection matrix, respectively. **b**_1_ and **b**_2_ denote the first and second bias, respectively.

Generally, the cross-modal transformer enables one modality to receive information from another modality. Specifically, EEG signals continue updating the sequence and are transformed to a different set of key/value pairs to interact with the corresponding auditory stimulus through the cross-modal transformer and vice versa. Therefore, we obtained the outputs from cross-modal attention transformer as follows: ${\text{O}}_{E\to A}^{\left[N\right]}$, ${\text{O}}_{E\to B}^{\left[N\right]}$, ${\text{O}}_{A\to E}^{\left[N\right]}$, and ${\text{O}}_{B\to E}^{\left[N\right]}$. The CMAA model is composed of a stack of *N* = 5 layers in this study.

### 2.4. Output Layer and Classification

To avoid any information loss in data compression, we redefined the simulation regression problem as a classification problem in this paper. Our CMAA model directly chooses the attended speaker by selecting the closest audio stream based on the cosine similarity (Herff et al., 2019) of the corresponding EEG features. Such operation does not contain any additional learning parameters, and it is intuitive that the inner product measures the cosine similarity between audio and EEG features. The cosine similarity between vectors X and Y is defined as:

(9) $$\begin{array}{l} \text{similarity}\left(\text{X},\text{Y}\right)=\frac{\text{X}\cdot \text{Y}}{\left\|\text{X}\|\|\text{Y}\right\|}\\ \;=\frac{\sum_{i=1}^{n}X_{i}Y_{i}}{\sqrt{\sum_{i=1}^{n}X_{i}^{2}}\sqrt{\sum_{i=1}^{n}Y_{i}^{2}}} \end{array}$$

Therefore, the cross-modal similarity between EEG and audio of speaker A is obtained by computing their cosine distance as $\text{similarity}\left({\text{O}}_{E\to A}^{\left[N\right]},{\text{O}}_{A\to E}^{\left[N\right]}\right)$. Similarly, we can obtain the cross-modal similarity between the EEG and audio of speaker B as $\text{similarity}\left({\text{O}}_{E\to B}^{\left[N\right]},{\text{O}}_{B\to E}^{\left[N\right]}\right)$. Previous research indicates that when two speech streams are presented simultaneously, neural activity shows stronger correlation with the temporal envelope of the attended speech stream than with the unattended speech (Ding and Simon, 2012; Mesgarani and Chang, 2012; O'Sullivan et al., 2015). Therefore, the speaker with higher similarity will be classified as the attended speaker, while the speaker with lower similarity will be classified as the unattended speaker. Here we employed multiple fully connected (FC) layers to choose the attended speaker in a binary decision. Specifically, the first layer contains T neurons with ReLU activation function. The second layer contains two (output) neurons with sigmoid activation function and weighted cross-entropy as the loss function.

### 2.5. Dataset and Setting

In this paper, experiments were carried out on an EEG and audio dataset for auditory attention decoding (Fuglsang et al., 2017, 2018), recorded at the Technical University of Denmark (DTU), and thus referred to as the DTU dataset; 64-channel EEG data were recorded at a sample rate of 512 Hz using a BioSemi Active system following the electrode locations of the international 10/20 system. The auditory stimuli in the DTU dataset were recorded at a sample rate of 48 kHz and comprised a male and a female speaker simultaneously speaking in simulated rooms with different degrees of reverberation. Specifically, recordings from two speakers in an anechoic room are referred to as being under anechoic conditions. The two concurrent speech streams were presented to subjects at 65 dB using loudspeakers, with distances of 2.4 m and positioned at ± 60° along the azimuth direction. Recordings from two target speakers corrupted by 6 additional background speakers (3 male, 3 female) in a reverberant room are referred to as being under reverberant conditions. According to the clarity, which is defined as the ratio of the direct 80-ms sound energy to the remaining energy (Fuglsang et al., 2017), mild reverberation ranges between *C*_80,63 *Hz*_ = 5.7 dB and *C*_80,63 *Hz*_ = 7.4 dB, and high reverberation ranges between *C*_80,63 *Hz*_ = 6.7 dB and *C*_80,63 *Hz*_ = 9.7 dB.

EEG data from 18 subjects were collected. All participants were students with self-reported normal hearing and no history of neurological disorders. Each subject listened to 60 trials in total, and each trial contained auditory stimuli with a duration of 50 s. Prior to each trial, the subjects were told to attend to one speech stream and ignore the other speech stream. After each trial, subjects were required to answer a multiple-choice question related to the content of the attended speech stream. The position of the target streams and the gender of the speaker were randomized across the trials.

### 2.6. Data Processing

EEG signals were first processed to filter out 50 Hz line noise and harmonics (de Cheveigné and Arzounian, 2018). Eye artifacts were subsequently removed using a joint decorrelation framework (de Cheveigné and Parra, 2014). Then, the data of each channel were re-referenced to the average response of the mastoid electrodes. All the EEG data were bandpass-filtered between 2 and 32 Hz with a finite impulse response (FIR) filter and subsequently downsampled to 70 Hz. The frequency range was chosen based on the previous non-linear AAD studies (de Taillez et al., 2017; Deckers et al., 2018; Vandecappelle et al., 2021). Finally, the EEG data channels were normalized to ensure zero mean and unit variance for each trial.

Previous studies have shown that the power-law compression model resembles the non-linear transformation process of the speech streams in the human auditory system that is effective in the AAD experiment (Biesmans et al., 2016). In brief, a gammatone filterbank ranging from 150 to 4,000 Hz was used to filter the auditory stimuli into subbands. Each subband was further processed with a power-law compression with an exponent of 0.6. The subband envelopes were then added to generate a broadband envelope, which was filtered with the same filter as used for the EEG recordings and then downsampled to 70 Hz to match the EEG data (Deckers et al., 2018; Vandecappelle et al., 2021), denoted as H-LP in Figure 1. Finally, the stimulus amplitudes in each speech stream within each trial were normalized to have the same RMS intensity.

### 2.7. Training and Evaluation

The CMAA model was evaluated against a reference baseline, and the performance characteristics of AAD for 5, 2, and 1 s were reported, with the decoding accuracy defined as the percentage of correctly classified decision windows. Data of each subject were randomly divided into training (80%), validation (10%), and test sets (10%). For each partition, data segments were generated with a sliding window, which we call a decision window, with an overlap of 50%.

We trained the CMAA model for 200 epochs, and adopted the cross-entropy loss function as the cost function in the adaptive moment estimation algorithm (Adam) (Kingma and Ba, 2014). The learning rate was set to 1×10^−4^. All hyperparameters given above were determined by running a grid search over a set of reasonable values. Performance during this grid search was measured on the validation set.

To capture the general performance of the CMAA, the reported accuracy for each subject is the average accuracy of 10 different testing runs of the model, each with a different (random) initialization.

## 3. Results

In this study, we systematically investigated the effectiveness of cross-modal attention-based AAD. We studied the effect of the decision window size and acoustic conditions through comprehensive experiments.

Additionally, we performed experiments on the DTU dataset to benchmark the proposed framework against the state-of-the-art baseline. The CNN-based AAD model in (Deckers et al., 2018) was reimplemented on the DTU dataset for comparison since it showed state-of-the-art results on the AAD tasks. During training, the CNN network is optimized to predict the correct label, i.e., 0 or 1, which represents the attended speaker. We note that the CNN model in our study focused on processing the CSP-enhanced EEG data. Briefly, the CNN architecture includes a convolution layer [66–9], an average pooling and two FC layers (Input: 10, hidden: 10, output: 2). The ReLU activation function is used after the convolution step, and the sigmoid activation function is used after each FC layer. The loss function is the weighted cross-entropy loss. To train the CNN network, the initial learning rate was 0.1 and was halved successively after 10, 25, and 40 training epochs.

### 3.1. Decoding Performance

**Figure 4:**
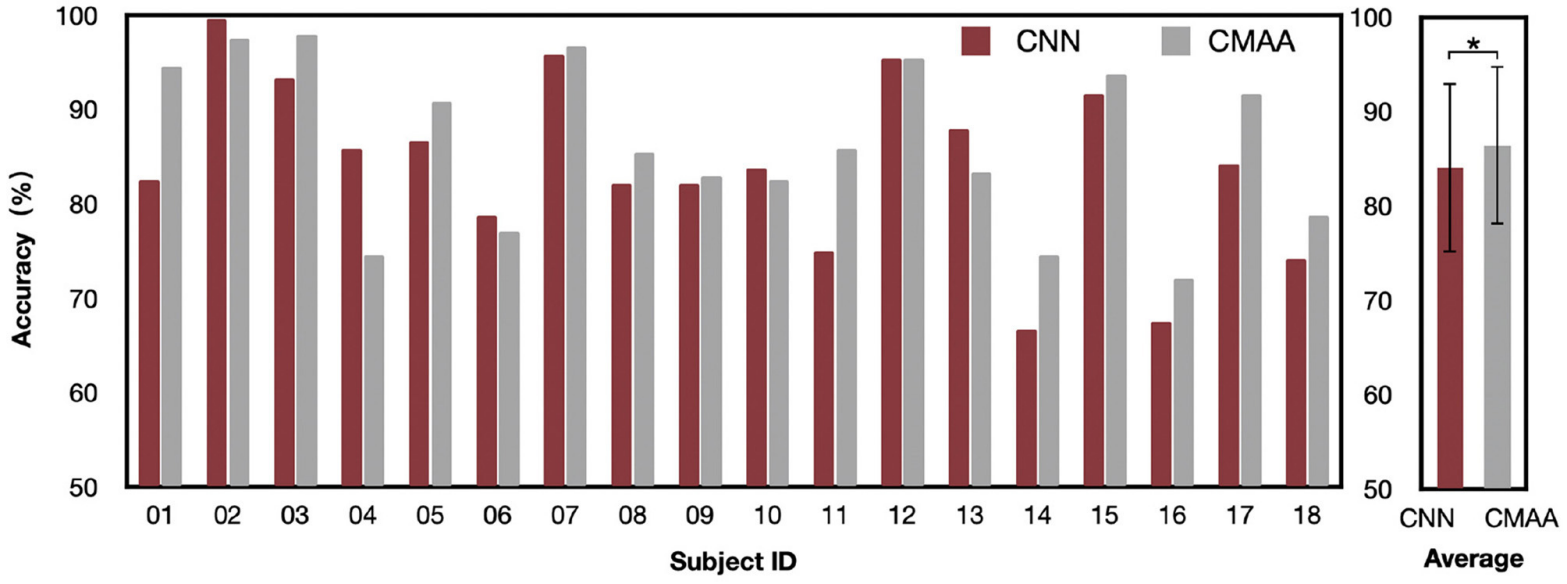
Auditory attention detection accuracy of the CMAA and CNN model across all subjects under anechoic conditions with the 2-s decision window. Significance was calculated using paired *t*-test (*p <0.05).

We report the AAD accuracy of the CMAA and CNN models across all subjects in subject-dependent scenario in Figure 4. For the 2-s decision window, the CNN model obtains an average accuracy of 84.1%, with a standard deviation of 9.04. The proposed CMAA model achieves better AAD performance, with an average accuracy of 86.4% (standard deviation or SD: 8.43). The percentages of the subjects who achieved 90% classification performance are 44.4% (8 of 18) and 27.8% (5 of 18) for the CMAA and CNN model, respectively. Additionally, the classification accuracy for all participants was greater than 70%, indicating that the proposed CMAA model may be a promising solution for detecting auditory attention in a cocktail party scenario.

Statistical analyses were performed using SPSS 24.0 (SPSS Inc., Chicago, IL, United States). All outcomes were inspected for normal distribution using the Kolmogorov-Smirnov test, prior to selection of appropriate statistical tests. A significance level of *P* <0.05 was used for all the analyses. The AAD performance of the CMAA model significantly outperforms that of the CNN model (paired *t*-test: *p* = 0.03), which validates the contribution of the cross-modal attention mechanism.

### 3.2. Effect of the Decision Window Length

**Figure 5:**
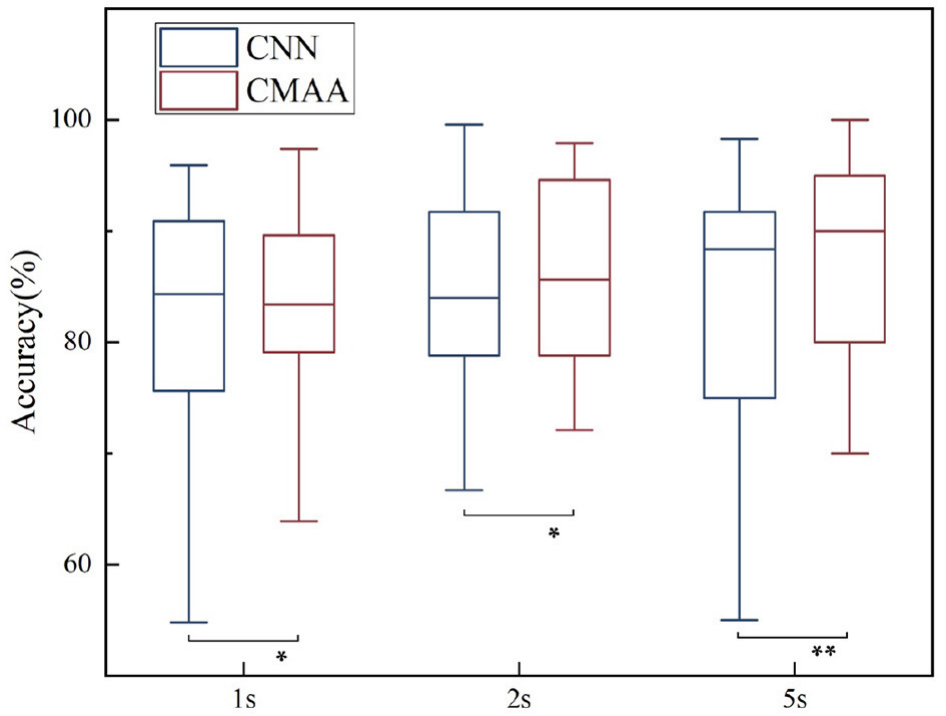
Auditory attention detection performance of the CMAA model for three different decision windows. CNN decoding model is shown as baseline. Significance was calculated using paired *t*-test (*p <0.05, **p <0.01).

To realize real-time AAD, our study concentrates on shorter decision windows. Specifically, we compare the AAD performance of the CMAA model for the decision window sizes of 1, 2 and 5 s, as illustrated in Figure 5. Consistent with previous findings (Fuglsang et al., 2017; Wong et al., 2018), the best decoding performance is obtained with the 5-s decision window (mean: 87.6%, SD: 8.86), followed by the 2-s decision window (mean: 86.4%, SD: 8.43) and 1-s decision window (mean: 82.8%, SD: 8.89). This result may be because shorter decision windows contain less information and therefore result in poorer performance than the longer decision windows (Miran et al., 2018; Das et al., 2020).

It is important to note that in the case of operating at low latency settings, our proposed CMAA method consistently outperforms the state-of-the-art methods. Specifically, the CMAA model is capable of boosting the performance of a non-linear AAD decoder, leading to 1.1, 2.3, and 2.4% performance gains for the 1-, 2-, and 5-s decision windows, respectively. These results demonstrate the promising potential of the proposed CMAA method for the practical implementation of real-time AAD.

### 3.3. Effect of Acoustic Conditions

**Figure 6:**
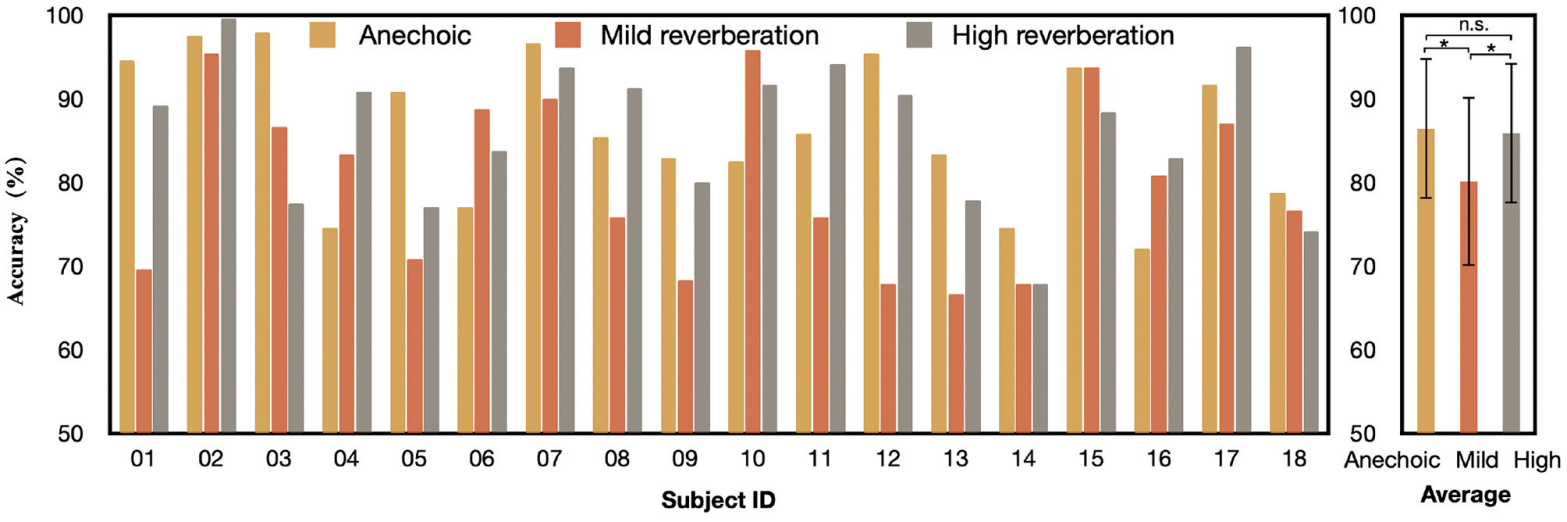
Auditory attention detection accuracy of the CMAA model across all subjects under three different acoustic conditions with the 2-s decision window. Significance was calculated using paired *t*-test (*p <0.05, n.s., no significance).

To understand how the proposed CMAA model behaves under different acoustic conditions, we also trained and tested the CMAA under three listening conditions, namely, anechoic, mild reverberation, and high reverberation settings. These results are reported in Figure 6. For the 2-s decision window, the CMAA model obtained the best decoding performance under anechoic conditions (mean: 86.4%, SD: 8.43), followed by high reverberation conditions (mean: 85.9%, SD: 8.39) and mild reverberation conditions (mean: 80.1%, SD: 10.01), or an average of 84.1%. The paired *t*-test provided evidence of a small, statistically significant difference between the anechoic and mild reverberation conditions (*p* = 0.04). Moreover, we found that there is no statistical difference between the AAD accuracy of the anechoic and high reverberation conditions (paired *t*-test, *p* = 0.41). One explanation could be that attention to one specific speaker becomes harder under high reverberation condition, and consequently demands more effort from the subject (Das et al., 2018). Findings of previous fMRI (Zekveld et al., 2006) and ECoG (Golumbic et al., 2013) research have also shown that brain regions involved in top-down processing supplementing speech comprehension to be more active when the speech was less intelligible. With the improvement in signal-to-noise ratio of the neural responses, it is possible to realize relatively accurate attention decoding under challenging auditory conditions, such as high reverberation condition. It also consistent with the findings by Fuglsang et al. (2017) that percentage of correctly answered comprehension questions related to the content of the attended stories drops in mild reverberation in comparison with anechoic and high reverberation conditions. Considering that the answers served as an indicator of whether the subjects attended the target talker and whether the speech was comprehensible in the different listening conditions, it makes sense that the AAD performance of CMAA decreases slightly in mild reverberation condition.

In general, the AAD performance of the proposed CMAA model is still better than 80% in different acoustic environments. The experimental results suggest that the CMAA can achieve robust detection accuracy of auditory attention decoding even in the presence of real-world reverberation.

## 4. Discussion

We present a CMAA model that dynamically adjusts the interaction between audio and EEG features in order to improve the low-latency AAD performance. To the best of our knowledge, this is the first study to apply the cross-modal attention mechanism, which can adapt streams from one modality to another (e.g., EEG → audio), in the EEG-based AAD tasks. Using this mechanism, we hope to build the correlation between auditory stimuli and EEG responses, thus detecting the attention activities manifested in brain signals. The proposed CMAA model has realized high AAD accuracy even with 1-s decision window. Additionally, the experimental results demonstrate that the proposed CMAA can detect the attended speaker from a mixture of two speakers and is stable against varying amounts of reverberation. Generally, the low-latency and noise-robust CMAA model paves a way for developing new neurofeedback training paradigms that require EEG-based attention decoders (Kim et al., 2021).

To further validate our method and understand the functioning of the cross-modal attention mechanism, we next compare the proposed CMAA model with other competing models in the literature.

### 4.1. Comparative Study

We start by comparing CMAA model with other linear models reported in the literature in a subject-dependent test. Wong et al. (2018) reported the AAD performance on the same DTU dataset with a linear model. Table 1 shows the average decoding accuracies across all subjects for individual methods. The AAD accuracies of the linear model with low latency are fairly low, while our proposed CMAA model can obtain an average accuracy over 80%, even for the 1-s decision window. These results demonstrate that our method significantly outperforms the other reported linear mapping methods on the same dataset with a large margin (*p* <0.01). The better AAD performance of the CMAA model also validates that the correlations of the audio and EEG signals can be captured by the proposed cross-modal attention mechanism.

**Table 1:** Auditory attention detection accuracy (%) in a comparative study of different models on the same DTU dataset with different window lengths under anechoic conditions.

**Model**	**Decision window**		
	**1 s**	**2 s**	**5 s**
Linear (Wong et al., 2018)	55	61	70
CNN^*^	81.7	84.1	85.2
CMAA	82.8	86.4	87.6

* *Here, we reimplement the CNN model in Deckers et al. (2018) with our experiment setup for comparison*.

It is noted that non-linear models show much better performance than linear models, particularly in low-latency settings (de Taillez et al., 2017; Deckers et al., 2018; Ciccarelli et al., 2019; Vandecappelle et al., 2021). Since the other reported non-linear models are reported on different datasets, a direct comparison with CMAA is not straightforward. We therefore reimplement the CNN-based AAD model in (Deckers et al., 2018) to process the CSP-enhanced EEG data of the DTU dataset. As shown in Table 1, our proposed CMAA method significantly outperforms that reported in (Deckers et al., 2018) with consistent improvements in AAD accuracy with different decision windows (*p* = 0.01).

To summarize, the performance of the proposed CMAA compares favorably with that of the state-of-the-art AAD models on the public DTU dataset in low latency settings. The results support the suitability of the proposed CMAA method for developing new neurofeedback (or perceptual) training paradigms.

### 4.2. Contributions of Cross-Modal Attention to Auditory Attention Detection

Our proposed CMAA model yields competitive performance compared with the existing AAD models. To obtain better insight into the underlying reasoning processes that CMAA learns to perform, we study the visualizations of the attention distributions produced by the cross-modal attention during its iterative computation.

**Figure 7:**
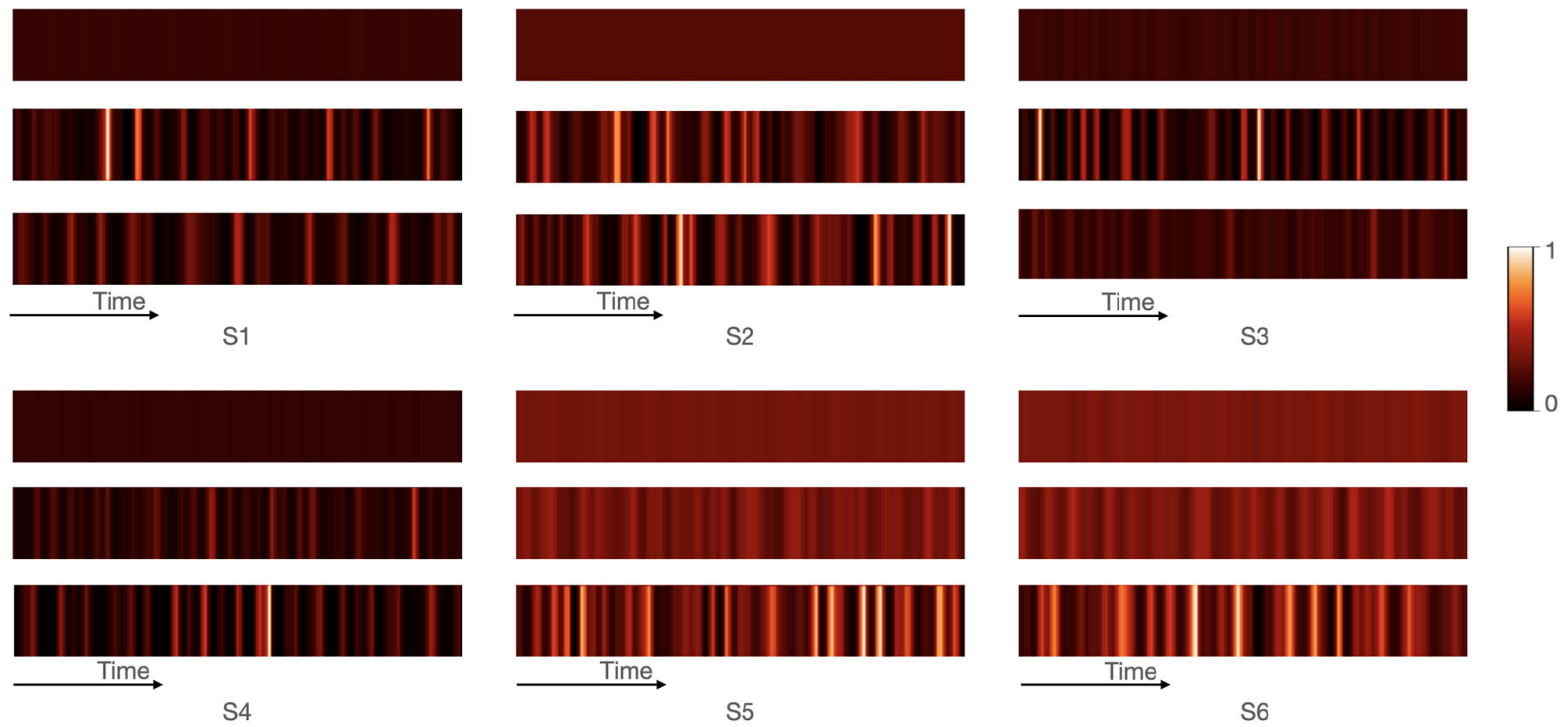
Visualization of the cross-modal attention module from EEG to audio. Six samples were randomly chosen and defined as S1–S6. For S1–S3, the attended speaker is speaker A, while the attended speaker is speaker B in S4–S6. Each sample contains three attention matrices. The first row depicts attention weights before cross-modal attention model. The second and third row represents the attention weights of EEG that are adjusted according to the audio of speaker A and speaker B, respectively. In each matrix, the weights of all EEG channels are aggregated to presents the attention weights of EEG across time. The color of the cells describes the weight with lighter color corresponding to larger weight.

We take E2A attention as an example in which the weights of the EEG signals were adaptively adjusted based on the audio attention vector. The cross-modal attention weights show the most relevant EEG inputs for each audio stream, as illustrated in Figure 7. The weights of multi-channel of EEG signals were aggregated in each matrix. A whiter cell indicates higher attention, while a darker cell indicates lower attention. In the first row, it is observed that EEG inputs have same weights across time before they were processed by cross-modal attention module. The second row represents the attention weights of EEG that are adjusted according to the audio of speaker A, while the third row represents the attention weights of EEG that are adjusted according to the audio of speaker B. These results demonstrated that the cross-modal attention model can pay attention to more relevant EEG inputs for each audio. Brain activities related to attention also show similar mechanisms to that of humans who confine their attention to the behaviorally relevant information and inhibit the processing of irrelevant information (Zanto and Gazzaley, 2009; Foxe and Snyder, 2011; Vanthornhout et al., 2019). However, we are not aware of other studies on the AAD tasks, using both linear and non-linear models (Mirkovic et al., 2015; O'Sullivan et al., 2015; Van Eyndhoven et al., 2016; Deckers et al., 2018; Ciccarelli et al., 2019; Bednar and Lalor, 2020; Cai et al., 2020; Wang et al., 2020; Vandecappelle et al., 2021), which can emphasize more important and discriminative components of the EEG signal for the AAD based on the audio attention vector.

Additionally, examining the attention distributions not only help to provide a degree of interpretability for the proposed CMAA model but also present evidence for the classifications. As shown in Figure 7, the attended speaker is speaker A in S1–S3, while the attended speaker is speaker B in S4–S6. For S1–S3, our proposed CMAA model classified S1 and S3 correctly, while S2 was wrongly classified as speaker B. The visualizations show that the second row of S1 and S3 are much lighter than the third row, indicating that the attention weights of the EEG signals corresponding to the audio of speaker A are larger than the attention weights corresponding to the audio of speaker B. In comparison with S1 and S3, the attention distributions of the second and third row of S2 are similar, explaining the incorrect classification result. For S4–S6, which were classified as speaker B correctly by the proposed CMAA model, the second row is much darker than the third row, indicating that the attention weights of the EEG signals corresponding to the audio of speaker A are smaller than the attention weights corresponding to the audio of speaker B. These results indicated that the cross-modal attention has learned to capture the relevant parts of the auditory stimulus even with competing audio.

Overall, it is encouraging to find that the cross-modal attention mechanism can effectively adapt the EEG streams to audio streams by repeated reinforcing of the EEG features with those from the audio stimuli, or vice versa, thus improving the AAD performance. Our newly designed CMAA algorithm can dynamically modulate the interactions of EEG and audio in a cocktail party scenario. Compared with classic linear mapping, the CMAA model, which mimics human auditory attention (Mesgarani and Chang, 2012; Forte et al., 2017; Kaya and Elhilali, 2017; Obleser and Kayser, 2019), is a more advanced “decoding” strategy to realize robust real-time AAD.

### 4.3. Future Work

Throughout the paper, we assume that the clean audio of the speakers in a mixture are available; however, the access to clean sources is not realistic in real-world applications. The auditory stimuli must be extracted from acoustic mixtures as recorded by the acoustic applications such as hearing aids. Recently, some algorithms (Van Eyndhoven et al., 2016; Das et al., 2020) have been proposed to extract and denoise the auditory streams in a two-speaker acoustic scenario, relying on microphone array recordings from a binaural hearing aid. These sophisticated noise suppression systems can be integrated in our proposed model as a preprocessing module of auditory stimulus. We will further investigate the feasibility and effectiveness of this extension framework in future research.

## 5. Conclusion

AAD has attracted increasing interest for its potential application to hearing-aid design in the multiple competing speakers scenario. In this paper, we proposed a novel CMAA approach to detect the attended speakers in a cocktail party scenario. The CMAA model can dynamically adjust the interaction between the EEG responses and auditory stimuli and transform both the auditory stimulus and the EEG response. The experimental results on a benchmark dataset indicate that our proposed CMAA method significantly outperformed the previous subject-independent as well as conventional subject-dependent approaches. Moreover, data visualization and the aforementioned results suggest that the correlations between audio and EEG can be captured by the cross-modal attention mechanism in the CMAA model. In conclusion, our newly designed CMAA approach paves a way for real-time and robust AAD even in complex acoustic environments.

## Data Availability Statement

Publicly available datasets were analyzed in this study. This data can be found here: https://zenodo.org/record/1199011#.X_xXw2QzZ6J. The code for the proposed model is provided in https://github.com/SCUT-IEL/CMAA.

## Author Contributions

SC and PL contributed to the design of the experiments, the analysis and interpretation of data, and the writing of the manuscript. ES contributed to the implement of the experiments. LX contributed to the revision of the manuscript. All authors contributed to the article and approved the submitted version.

## Conflict of Interest

The authors declare that the research was conducted in the absence of any commercial or financial relationships that could be construed as a potential conflict of interest.
